# Unveiling the Wheat Microbiome under Varied Agricultural Field Conditions

**DOI:** 10.1128/spectrum.02633-22

**Published:** 2022-11-29

**Authors:** Sarika Jaiswal, Bharti Aneja, Jaisri Jagannadham, Bharati Pandey, Rajender Singh Chhokar, Subhash Chander Gill, Om Parkash Ahlawat, Anuj Kumar, U. B. Angadi, Anil Rai, Ratan Tiwari, Mir Asif Iquebal, Dinesh Kumar

**Affiliations:** a Division of Agricultural Bioinformatics, ICAR-Indian Agricultural Statistics Research Institute, New Delhi, India; b ICAR-Indian Institute of Wheat and Barley Research, Karnal, Haryana, India; c Centre for Bioinformatics & Computation Biology, Ashoka University, Rajiv Gandhi Education City, Sonepat, Haryana, India; d Department of Biotechnology, School of Interdisciplinary and Applied Sciences, Central University of Haryana, Mahendergarh, Haryana, India; USDA—San Joaquin Valley Agricultural Sciences Center

**Keywords:** wheat, 16S rRNA, metagenomics, microbiome, CAZy, microbial diversity

## Abstract

Wheat being the important staple food crop plays a significant role in nutritional security. A wide variety of microbial communities beneficial to plants and contributing to plant health and production are found in the rhizosphere. The wheat microbiome encompasses an extensive variety of microbial species playing a key role in sustaining the physiology of the crop, nutrient uptake, and biotic/abiotic stress resilience. This report presents wheat microbiome analysis under six different farm practices, namely, organic (Org), timely sown (TS), wheat after pulse crop (WAPC), temperature-controlled phenotyping facility (TCPF), maize-wheat cropping system (MW), and residue burnt field (Bur), using 16S rRNA sequencing methodology. The soil samples collected from either side of the wheat row were mixed to get a final sample set for DNA extraction under each condition. After the data preprocessing, microbial community analysis was performed, followed by functional analysis and annotation. An abundance of the phylum *Proteobacteria* was observed, followed by *Acidobacteria*, *Actinobacteria*, and *Gemmatimonadetes* in the majority of the samples, while relative abundance was found to vary at the genus level. Analysis against the Carbohydrate-Active Enzymes (CAZy) database showed a high number of glycoside hydrolase genes in the TS, TCPF, and WAPC samples, while the Org, MW, and Bur samples predominantly had glycosyltransferase genes and carbohydrate esterase genes were in the lowest numbers. Also, the Org and TCPF samples showed lower diversity, while rare and abundant species ranged from 12 to 25% and 20 to 32% of the total bacterial species in all the sets, respectively. These variations indicate that the different cropping sequence had a significant impact on soil microbial diversity and community composition, which characterizes its economic and environmental value as a sustainable agricultural approach to maintaining food security and ecosystem health.

**IMPORTANCE** This investigation examined the wheat microbiome under six different agricultural field conditions to understand the role of cropping pattern on soil microbial diversity. This study also elaborated the community composition, which has importance in economic (role of beneficial community leading to higher production) and environmental (role of microbial diversity/community in safeguarding the soil health, etc.) arenas. This could lead to a sustainable farming approach for food security and improved ecosystem health. Also, the majority of the microbes are unculturable; hence, technology-based microcultivation will be a potential approach for harnessing other cultured microorganisms, leading to unique species for commercial production. The outcome of this research-accelerated work can provide an idea to the scientists/breeders/agronomists/pathologists under the mentioned field conditions regarding their influence over their crops.

## INTRODUCTION

The world population is projected to be around 9.5 billion by 2050, which demands a substantial increase in food requirement (1). One of the most pressing problems faced by the world is that it is progressing neither toward food sufficiency as per United Nations Sustainable Development Goals (SDG) target 2.1 nor toward the eradication of malnutrition as per SDG target 2.2 due to various reasons. According to the FAO, more than 720 million people worldwide suffered from hunger in 2020.

Wheat is the second most important staple food in India and plays a very significant role in ensuring the country’s food and nutrition security (2). The highly diverse composition of the soil microbial community affects plant fitness, productivity, and growth. The crop microbiome composition plays fundamental roles, including nutrient uptake, abiotic and biotic stress resilience, and plant defense (3). Each soil type harbors distinct microbial communities which are affected by several factors, such as soil physicochemical properties, crop type, agricultural practices, environmental factors, etc. These may have positive or negative impacts on crop health and productivity (4). Cropping patterns such as organic (Org) farming and cropping sequences have been known to enhance the crop yield, soil microbiome diversity, and soil organic carbon levels. Conversely, in monocropping or short-rotation crop systems, soil nutrient depletion and decreased yield are obviated. Therefore, to study the impact of the cropping patterns in wheat, we focused on the various field conditions.

Traditional methods of growing food crops are not sufficient to meet the food needs of the rapidly growing population. Although quite rewarding, the continuous cultivation of rice wheat has led to several problems related to soil health, such as decreases in soil organic carbon content, poor infiltration rate, and poor water retention capacity. Besides these problems, the depletion of the water table, the incineration of residues, greenhouse gas emissions, waterlogging, and biodiversity loss, etc., have also turned out to be major challenges for sustainable development. To address these issues, the diversification of the rice-wheat system is the most needed change in the Indo-Gangetic plains, which are heavily dominated by this system. In recent years, corn has emerged as one of the potential alternatives to rice harvesting during the kharif season due to its high yield potential, short duration, and lower water requirements. Further, inclusion of pulses, such as summer moong as well as green manuring crops like dhaincha (*Sesbania* spp.) under rice-wheat crop rotation, has favorable effects. Yields of wheat grown after pulses are high, with increased protein content, high carbon sequestration, nitrogen transfer, and improved soil biodiversity. Therefore, the soil samples from these two cropping sequences warrant further study, motivating us to include them in the soil microbiome study.

Organic farming is a practice in its own right that does not require the use of synthetic fertilizers, pesticides, or growth regulators. It aims to improve soil biodiversity and fertility, control soilborne diseases, and promote sustainable production while protecting natural resources. Organic wheat has been found to have an improved grain zinc concentration; hence, it is in higher demand than wheat grown through conventional methods. Thus, we also collected soil samples from areas where organically cultivated wheat is grown.

Stubble (straw) burning is one a major problems; in this practice, farmers burn the crop residue to prepare the field for the next crop. This practice has severe adverse impacts on soil health. It leads to destruction of important soil microorganisms, soil nutrient loss, and an increase in crop vulnerability to diseases, thus making it an important area of research. Hence, we took soil samples from residue burnt fields (Bur) for our investigation. Among other limiting factors of wheat yield are the abiotic stresses, particularly, heat stress leading to substantial yield reduction under the changing climatic conditions. We also collected soil samples from a temperature-controlled phenotyping facility (TCPF), which is a low-cost structure for phenotyping for heat stress response under relative higher temperature compared to ambient, for the soil microbiome study.

Recently, next-generation sequencing (NGS)-based omics technologies have led to deeper insight into the microbial composition under various agriculture conditions. Studies of metagenomics of wheat from different geographic regions have been conducted extensively. Comprehensive research on microbiome diversity associated with wheat under diverse agricultural conditions, as an important aspect of plant growth, is still warranted. In this study, we evaluated the microbial functional diversity and composition differences across the six different agricultural practices and their comparison in organic and conventional farming systems. Our aim was to study the wheat-associated microbial community among all these agricultural practices to gain insight into the functional role played by each microbial species in plant-microbe interaction, which is important for sustainable crop production. We also aimed to study if each of the agricultural practices has any potentially unique microbial community signature and to assess the impact of wheat stubble burning on the soil microbial community. The high diversity/heterogeneity in the soil microbiome of wheat is good for plant health, while the loss of microbial community diversity suggests that changes in agricultural practices dynamically affect the soil microbial community over time.

## RESULTS

### Sample collection, data generation, and preprocessing of raw data.

**FIG 1:**
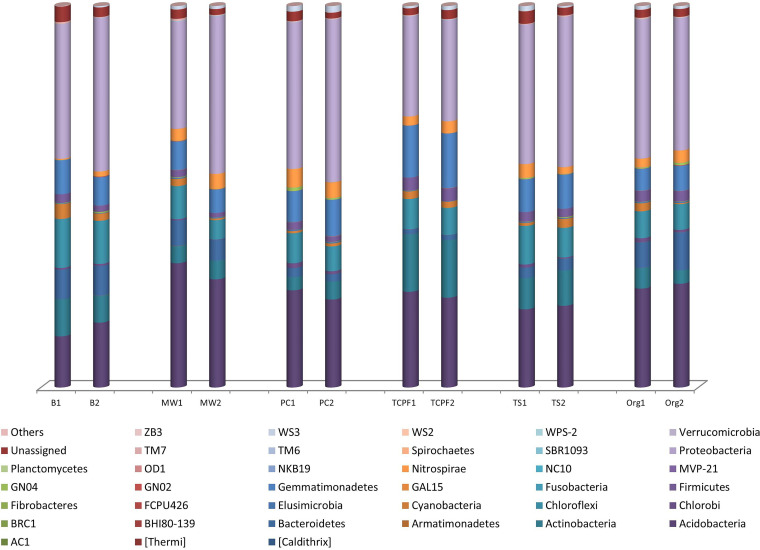
Microbial community analysis for biological replicates at phylum level. Bur, residue burnt field; MW, maize-wheat; WAPC, wheat after pulse crop; TCPF, temperature-controlled phenotyping facility; TS, timely sown.

DNA extracted from the soil samples was sequenced in duplicate to assess overall microbial community concordance. Raw paired-end reads from Illumina sequencing were processed to obtain better-quality reads. Table 1 describes the reads filtered at various steps. Before pooling of replicate samples, Welch’s *t* test statistical analysis was performed to derive the statistical significance at a *P* value of <0.05. For all the samples except Org, no significant difference was observed between replicates (*P* > 0.05). For these, replicate samples were processed individually and their results were pooled for analysis. Relative-abundance analysis at the phylum level showed *Proteobacteria* to be the most prevalent phylum, followed by *Acidobacteria*, in each replicate of six samples (Fig. 1). Further, six sets of the samples (TCPF versus Org, maize-wheat [MW] versus wheat after pulse crop [WAPC], timely sown [TS] versus TCPF, TS versus Bur, TS versus MW, and TS versus WAPC) were compared for the metagenomic analysis.

**TABLE 1:** Preprocessing results of sample reads generated from Illumina

Sample in replicates	Reads filtered out during preprocessing step		Total reads after all filtration steps for final analysis
	Unjointed reads during pairing of pair-end sequence	Quality filtering using Phred score (≥Q20)	
Organic 1	755,694	4,487	4,533,017
Organic 2	1,054,050	4,691	4,288,601
Maize-wheat 1	880,598	70,134	4,552,604
Maize-wheat 2	2,499,937	5,487	4,046,242
Timely sown 1	988,210	4,143	4,162,302
Timely sown 2	2,393,299	4,124	3,253,708
Wheat after pulse crop 1	1,995,915	4,525	3,708,622
Wheat after pulse crop 2	4,039,882	5,424	2,573,017
TCPF1	3,060,209	4,315	2,142,397
TCPF2	1,083,042	3,299	3,980,322
Residue burnt field 1	581,976	4,487	5,186,784
Residue burnt field 2	1,367,079	4,418	4,918,476

### Microbial community analysis.

**FIG 2:**
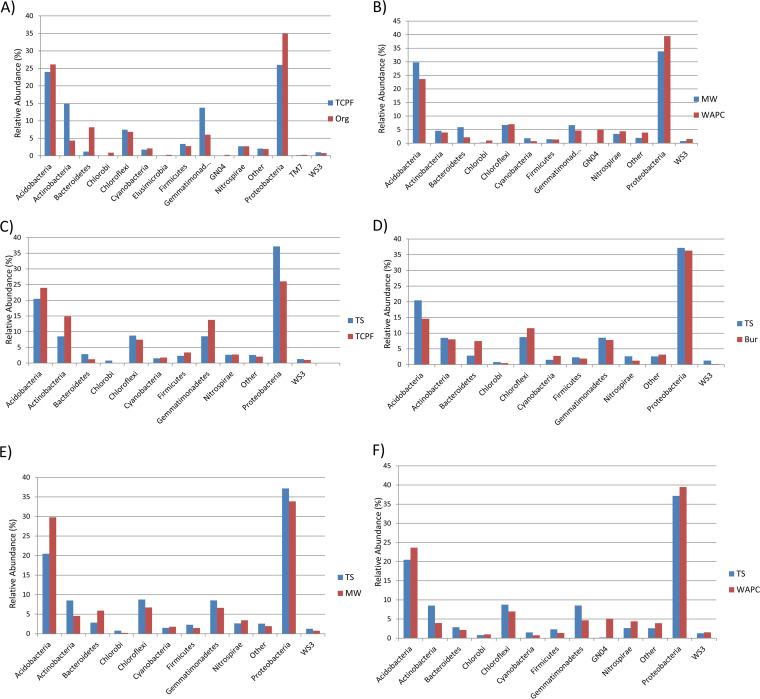
Phylum-level classification for all comparison sets. (A) TCPF and Org; (B) MW and WAPC; (C) TS and TCPF; (D) TS and Bur; (E) TS and MW; (F) TS and WAPC.

**FIG 3:**
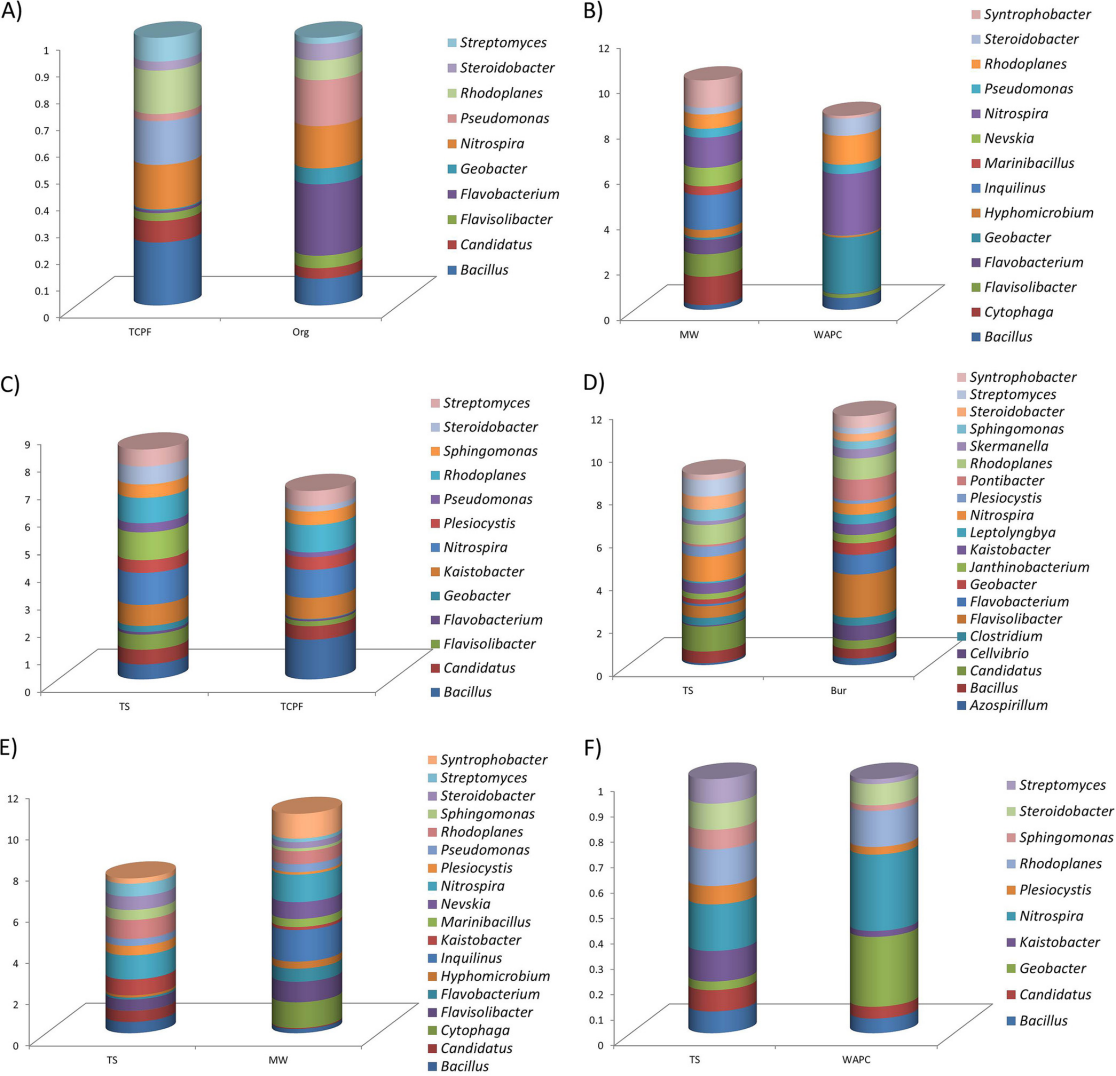
Genus level analysis in all the comparison sets. (A) TCPF and Org; (B) MW and WAPC; (C) TS and TCPF; (D) TS and Bur; (E) TS and MW; (F) TS and WAPC.

The high-quality preprocessed reads were clustered and classified into microbial operational taxonomic units (OTUs) in each sample. Low-abundance OTUs, i.e., OTUs with a count of 0, were excluded from further analysis. During taxonomic study of these samples, a few instances of unassigned taxon were observed, due to inadequate matches in the database. The overall microbial diversity of six sets of samples was analyzed up to the species level. Most of the samples were classified to the family level, while some were classified to the genus and species levels. The relative abundances at the phylum level and genus level in the all comparison sets are shown in Fig. 2 and 3, respectively. The phylum-level analysis revealed higher abundances of bacteria from the phylum *Proteobacteria* followed by *Acidobacteria* in all the comparison sets (Fig. 2). The relative abundance at the genus level was observed to vary among the six comparison sets. The highest relative abundances of the genera *Bacillus* (1% in both) and *Flavobacterium* (0.6 and 2.7%) were predicted in the TCPF and Org samples, respectively. The genus *Nitrospira*, which is reported to be the most abundant and ubiquitous nitrite-oxidizing bacterial (NOB) group (5), had the highest relative abundance and dominated in three comparison sets, namely, MW and WAPC (1.3% and 2.7%), TS and MW (1.1% and 1.3%), and TS and WAPC (1.1% and 2.7%). In the TS and TCPF comparison set, *Nitrospira* (1% in both) was found to be dominant genus in both. Moreover, in the TS and Bur set, *Nitrospira* (1.1 and 0.5%) and *Flavisolibacter* (0.5 and 2%) were found to be the prevalent genera in TS and Bur samples, respectively (Fig. 3). The relative abundance difference between samples at the phylum level is shown in Table S1 at http://webtom.cabgrid.res.in/Supplements/wheat_metagenome/. Table S2 shows the relative abundance difference between samples at the species level.

**FIG 4:**
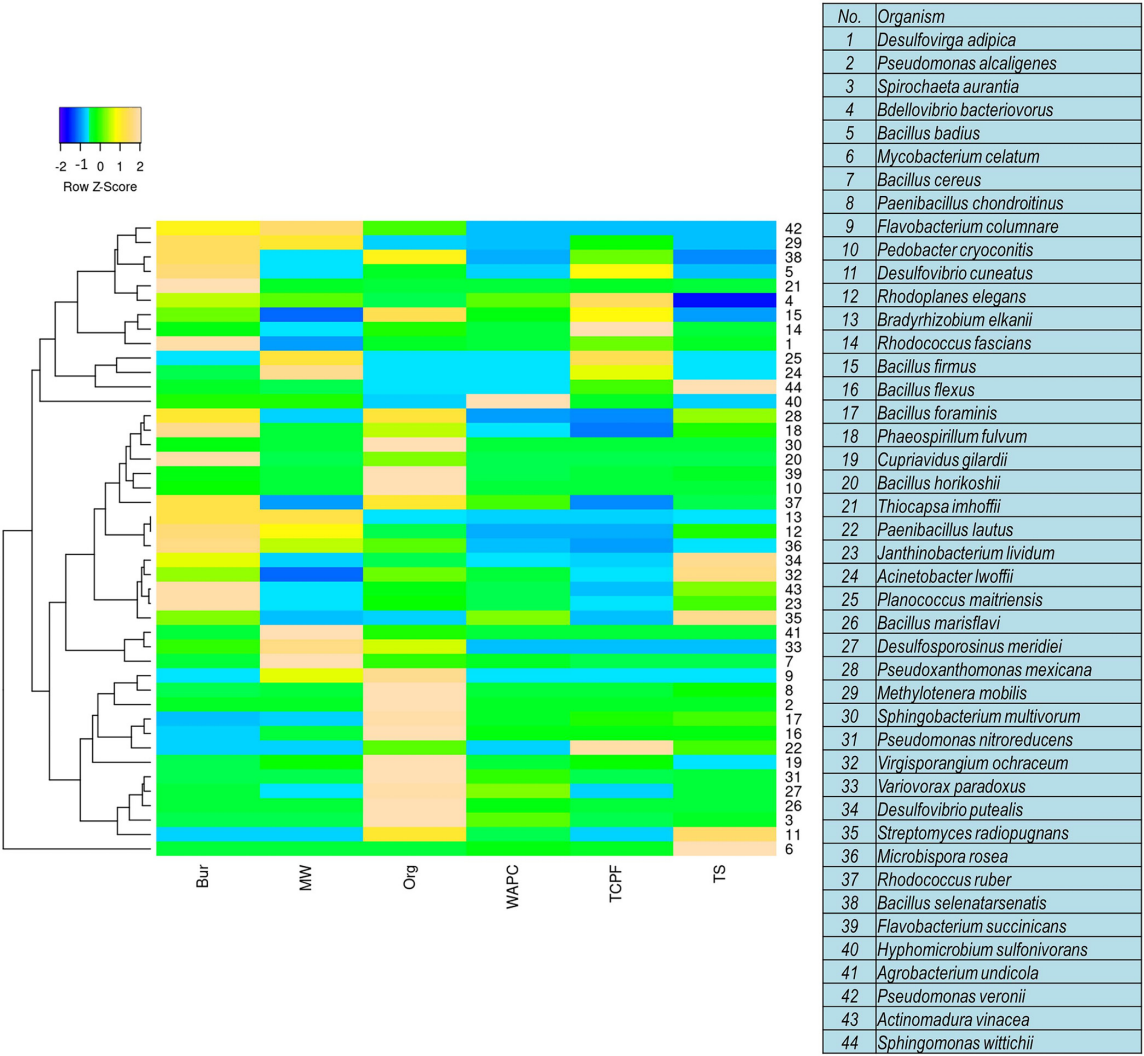
Heat map of Spearman’s rank correlation coefficients of the relative abundances of different microbes at the species level in different agriculture soil samples.

At the species level, the relative abundance of the microbial community was normalized, according to Spearman’s rank correlation clustering. The species-level heat map in Fig. 4 clearly indicates the presence of diversified organisms in all samples. Abundance profiles of community population were analyzed at the statistical level using Fisher exact *t* test. Based on *P* value analysis (*P* value < 0.05), it was observed that most of the bacterial genera were similar in comparison sets. Differences were recorded in the TCPF versus Org set, where *Acidobacteria* (*P* value, 6.09e−3) and *Flavobacterium* (*P* value, 0.027) showed significance difference. Similarly, in the TS versus MZ comparison set, differences were noticed for o_RB41 (*P* value, 6.03e−3) and o_11-24 (*P* value, 0.022). The comparison set TCPF versus TS showed differences for o_11-24 (*P* value, 0.03), while the comparison set TS versus Bur showed differences for o_11-24 (*P* value, 0.035).

The results show that the wheat microbial community at the phylum level consisted of different taxonomic groups, namely, *Actinobacteria*, *Acidobacteria*, *Proteobacteria*, *Gemmatimonadetes*, and *Bacteroidetes*, in the majority. Observation of these phyla between comparison sets based on the relative abundance difference of about 5% revealed the following: (i) *Actinobacteria* (14.8%) and *Proteobacteria* (34.9%) were dominant in the TCPF sample, while *Bacteroidetes* (8.1%) and *Gemmatimonadetes* (13.7%) showed dominance in the Org sample. (ii) In the MW and WAPC samples, *Acidobacteria* abundance (29.7%) was higher, while *Proteobacteria* (39.4%) and GN04 (5.09%) were abundant in the Org sample. (iii) *Actinobacteria* (14.8%) and *Gemmatimonadetes* (13.7%) were more abundant in the TCPF than in the TS sample. However, *Proteobacteria* (37%) was abundantly rich, by 11% in the TS sample as compared to TCPF sample. (iv) Similarly, with the TS and Bur comparison, *Bacteroidetes* (7.4%) and *Chloroflexi* (11.5%) were highly expressed in the Bur sample, with *Bacteroidetes* more abundant (7.4%) in the TS sample. (v) *Acidobacteria* (29.7%) and *Bacteroidetes* (5.9%) were dominant, with differences of about 10% and 4%, in the MW compared to the TS sample. Expression of *Actinobacteria* (8.5%) and *Proteobacteria* (37%) was high in the TS sample. (vi) Both *Actinobacteria* and *Gemmatimonadetes* were at a relative abundance of 8.5% in the TS sample in a study with the WAPC sample. Besides these phylum abundances already reported in other wheat microbiome studies, the distinct phyla like GN04, *Chloroflexi*, *Chlorobi*, *Nitrospirae*, and WS3 were found in our study (28).

**FIG 5:**
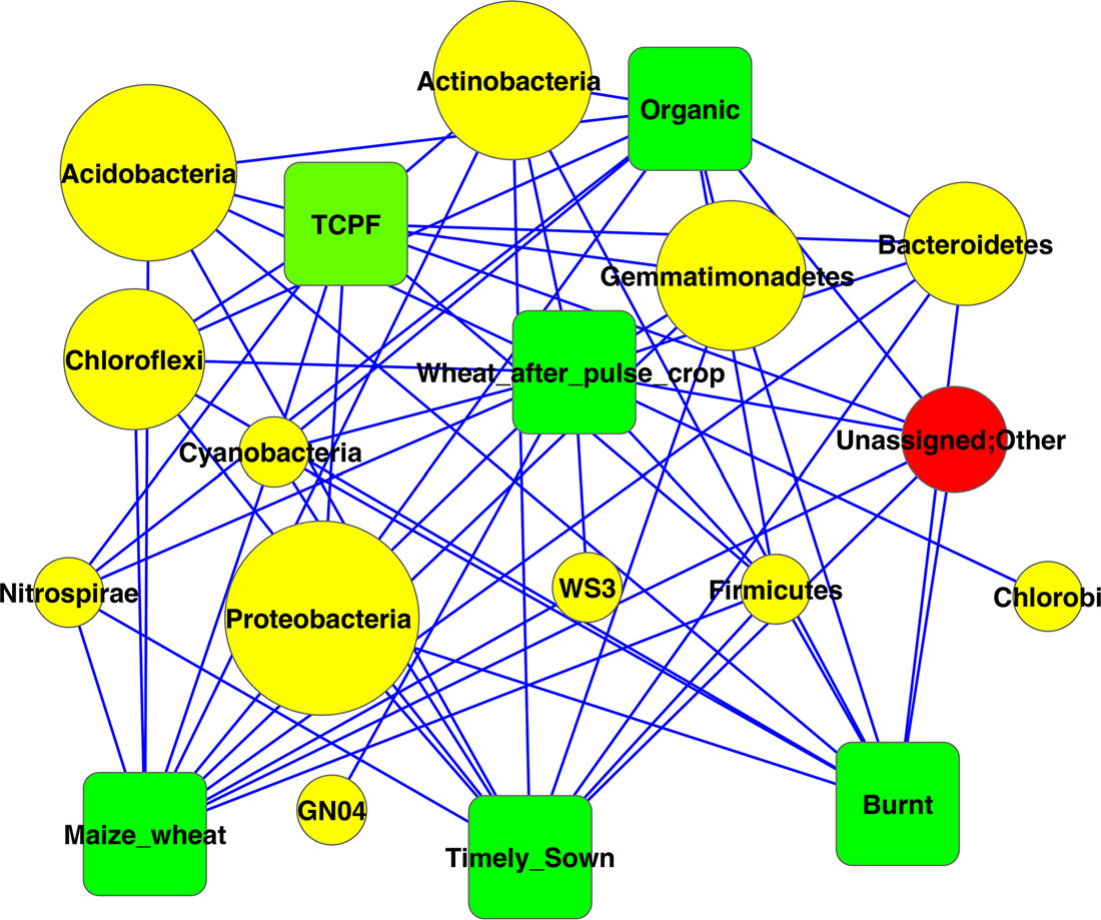
Network interaction of microbial taxonomic community at phylum level. Sizes of the nodes represent abundance percentage of the organism at the phylum level.

The strain-level network interaction analysis of the microbial taxonomic community was deciphered using Cytoscape (6) (Fig. 5). Larger nodes indicate dominance of the particular community. We found that *Proteobacteria* dominated the network, followed by *Acidobacteria* and *Actinobacteria* as the next common phyla in all six samples.

### Species richness.

**FIG 6:**
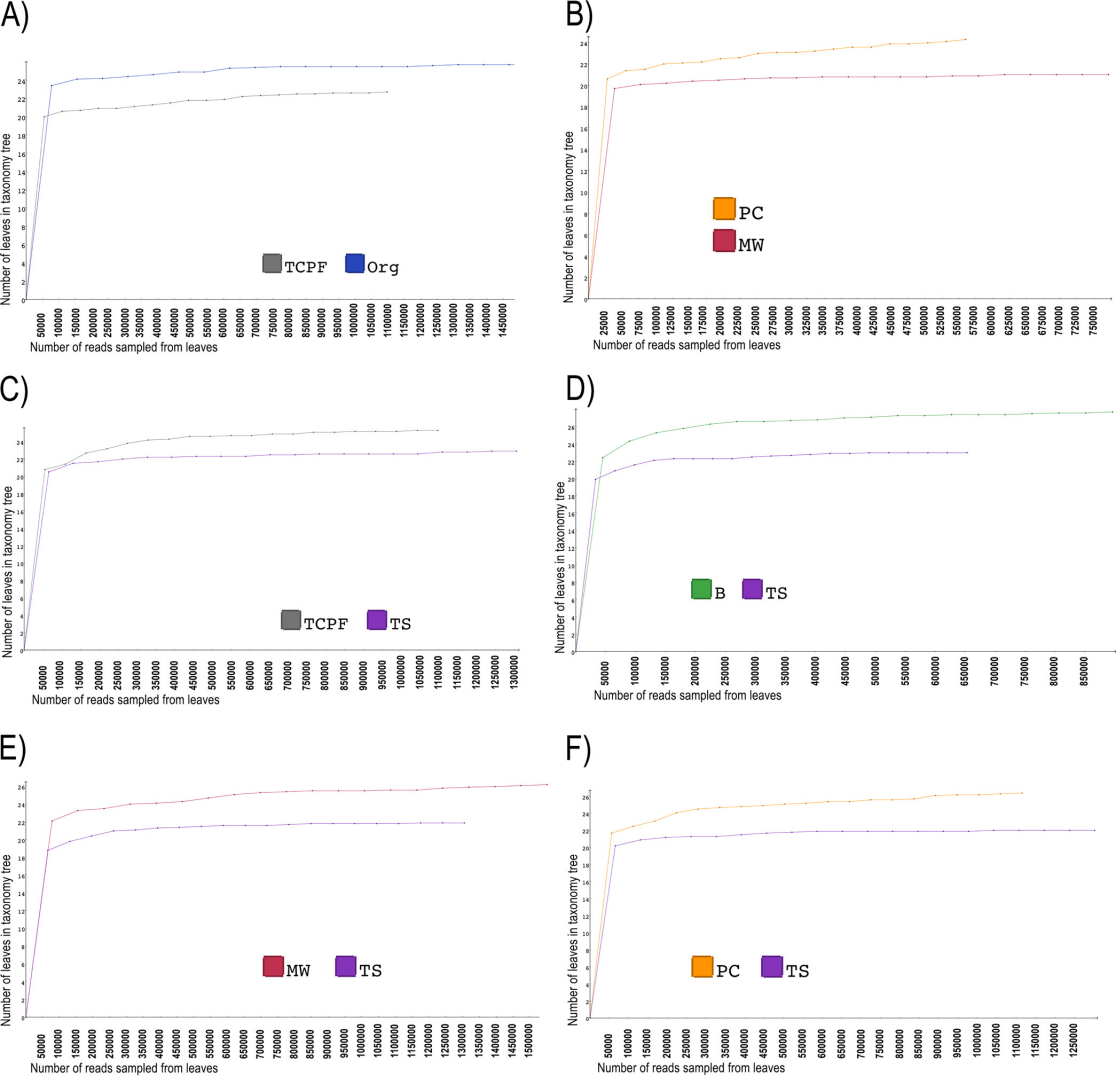
Rarefaction curve analysis in all sample sets.

**FIG 7:**
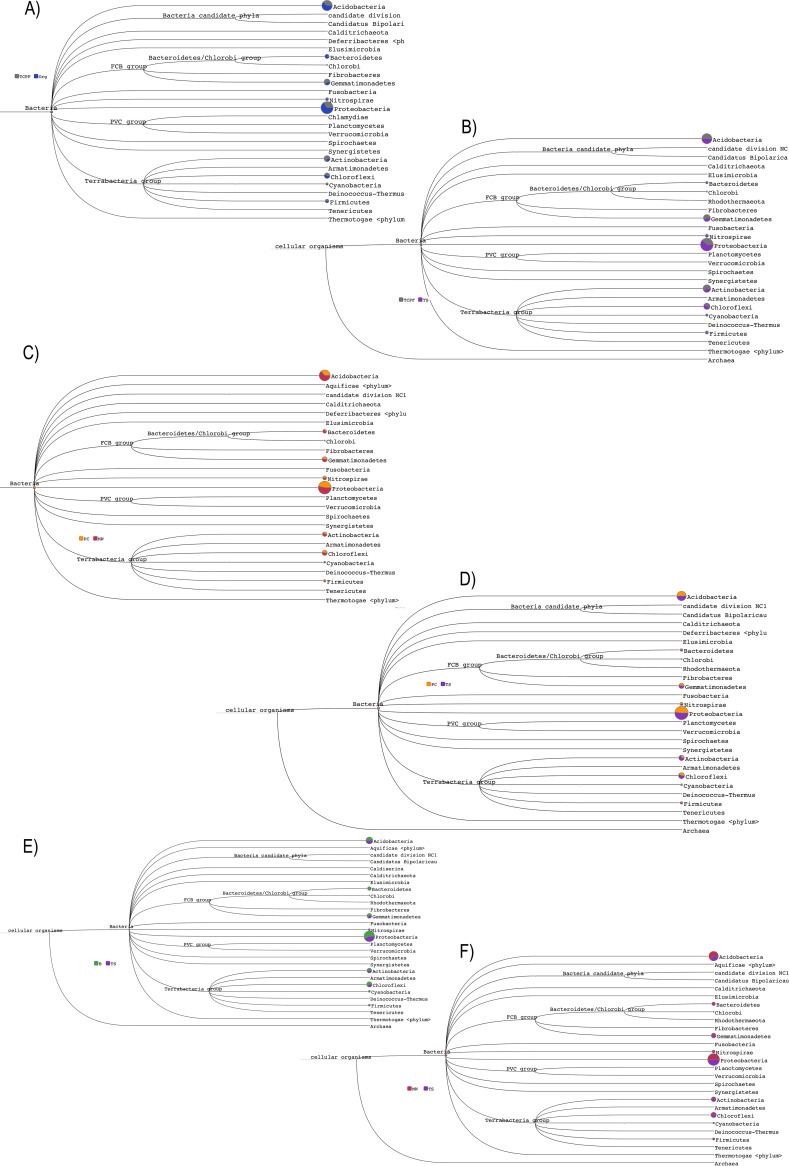
Phylogenetic tree prediction displayed for phylum level by MEGAN. FCB, *Fibrobacteres*, *Chlorobi*, and *Bacteroidetes*; PVC, *Planctomycetes*, *Verrucomicrobia*, *Chlamydiae*, and *Lentisphaerae*. Each circle represents a taxon, and the circle’s size is scaled logarithmically to reflect the number of reads immediately assigned to the taxon.

Species richness in each comparison set was estimated by rarefaction curve. Rarefaction curve plot analysis showed that maximum diversity was observed in TCPF, MW and TS datasets, where species richness was found to increase with number of sequence reads in these samples (Fig. 6). It was observed from rarefaction analysis that the curves for all the samples reached a plateau, indicating that the sequencing depth was sufficient. Based on phylum- and species-level analyses, phylogenetic study was implemented to decipher the hierarchy and relationship of taxonomic community for all the comparative sets. The relative abundance of the bacterial phylum is proportional to the size of the circle and revealed the dominance of *Proteobacteria* in all comparison sets (Fig. 7).

Included at the species level are the microbes specifically expressed in one of the sample sets on a predominant basis (i.e., based on their relative abundance). (i) For the TCPF and Org samples, Actinomadura vinacea (28%) was specific to the TCPF sample, whereas Flavobacterium succinicans (51%), Sphingobacterium multivorum (31.8%), Pseudomonas nitroreducens (22%), Paenibacillus chondroitinus (7.6%), and Janthinobacterium lividum (4.6%) were seen in the Org sample. (ii) For the MW and WAPC samples, Flavobacterium succinicans (9.8%) was abundant in the MW sample, while Hyphomicrobium sulfonivorans (1.4%) and Bacillus firmus (1.2%) were seen in the WAPC sample. (iii) For the TS and TCPF samples, Janthinobacterium lividum (7%) and Pseudoxanthomonas mexicana (4.2%) were abundant in the TS sample, while Rhodococcus fascians (1.6%) and Bacillus selenatarsenatis (1.5%) were seen in the TCPF sample. (iv) For the TS and Bur samples, Mycobacterium celatum (2.4%) and Phaeospirillum fulvum (2.2%) had high expression in the TS sample, while Phaeospirillum fulvum (6.4%) and Bacillus selenatarsenatis (2.9%) were predominant in the Bur sample. (v) For the TS and MW samples, Mycobacterium celatum (2.4%) and Phaeospirillum fulvum (2.2%) were in the majority in the TS sample, while Bacillus cereus (2.4%), Phaeospirillum fulvum (1.4%), and Flavobacterium columnare (1.25%) were rich in the MW sample. (vi) For the TS and WAPC samples, Pseudoxanthomonas mexicana (4.2%), Flavobacterium succinicans (2.7%), and Mycobacterium celatum (2.4%) were dominant in the TS sample and Hyphomicrobium sulfonivorans (1.4%) was seen in the WAPC sample.

### Diversity analysis.

**FIG 8:**
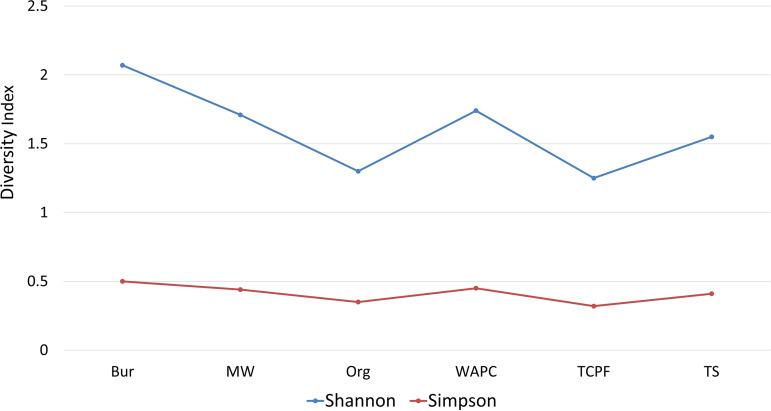
Diversity indices at Shannon and Simpson levels.

A comprehensive study on diversity indices was conducted to understand the existence of distinct organisms. Shannon-Wiener and Simpson indices measure the different types of species existing within samples. The results showed a high degree of diversity within the samples. The organic and TCPF samples showed less sample diversity (Fig. 8).

**FIG 9:**
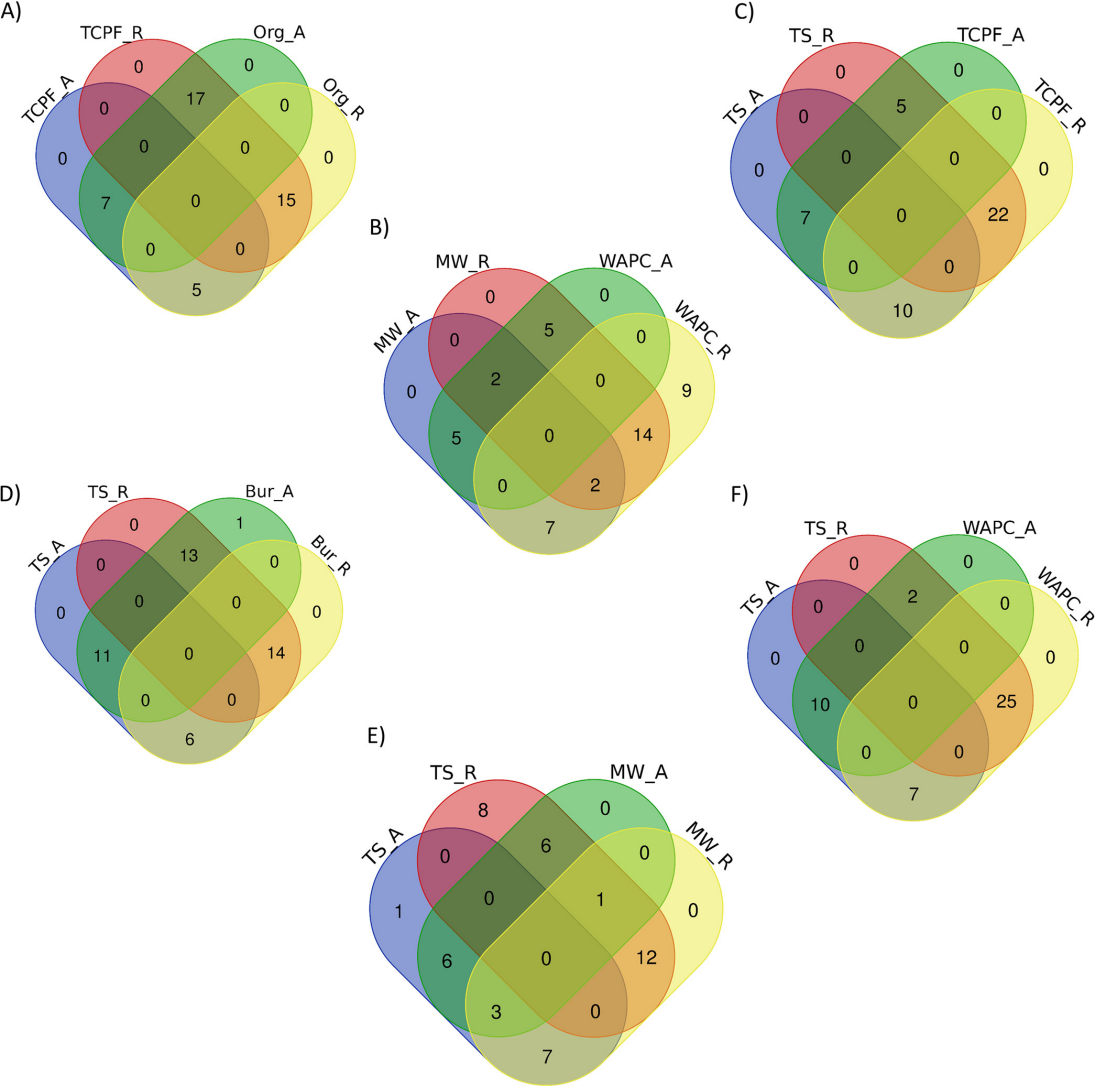
Comparison between abundant (A) and rare (R) species.

The species population can be classified into rare and abundant categories based on frequency of occurrence in a sample (7–9). Species with a very low representativeness in samples (abundance < 0.01) are classified as rare; otherwise, they are abundant. Similar analysis at the species level was performed that revealed that the percentages of rare species and abundant species ranged from 12 to 25% and 20 to 32% out of total bacterial species in all the compared sets of samples, respectively. The Venn diagram in Fig. 9 shows that most of the abundant species were present in both samples, with only a few being unique to one sample. Table S3 contains a detailed listing of rare and common species in all compared sample sets.

**FIG 10:**
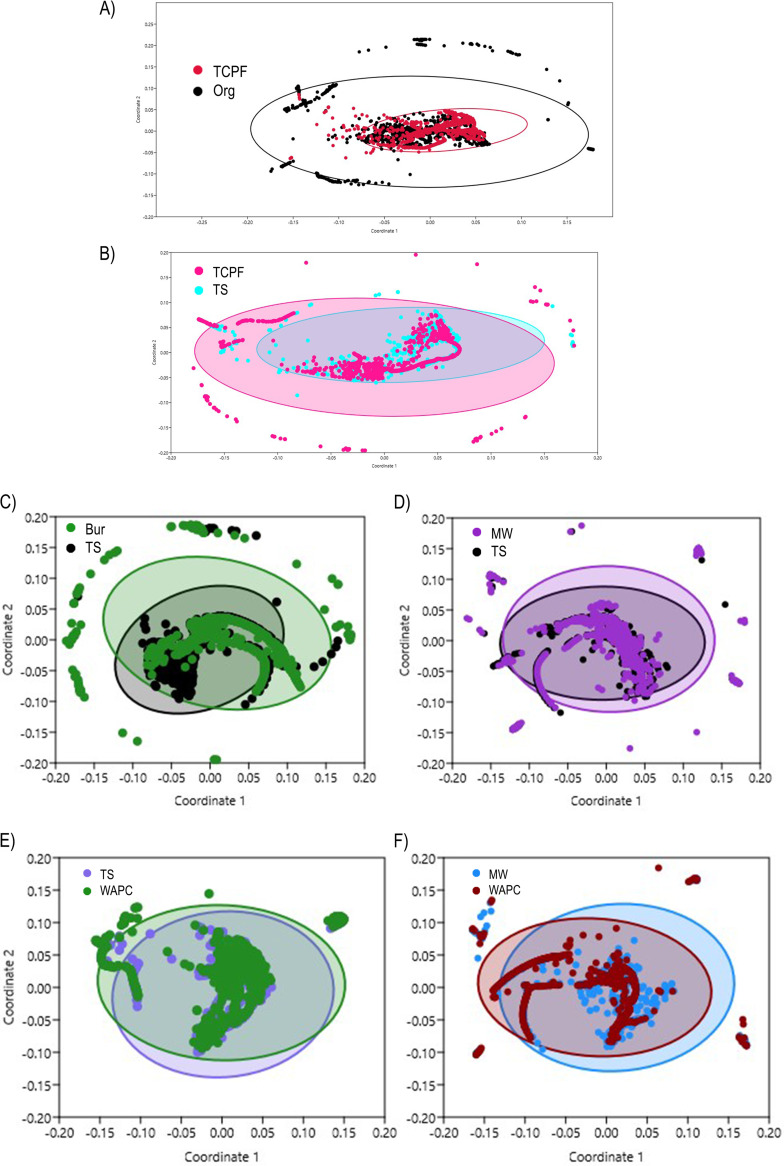
Bray-Curtis taxonomic similarities through nonmetric multidimensional scaling plot (A) TCPF vs. Org; (B) TCPF vs. TS; (C) Bur vs. TS; (D) MW vs. TS; (E) TS vs. WAPC; (F) MW vs. WAPC.

Compositional similarity between samples in all sets was determined from the Bray-Curtis similarity matrix for taxonomic species data. A multidimensional scaling plot using PAST (v3) delineates the similarity between and within samples (Fig. 10). Greater similarity was expressed through short linear distance and vice versa.

### Functional analysis and annotation.

**FIG 11:**
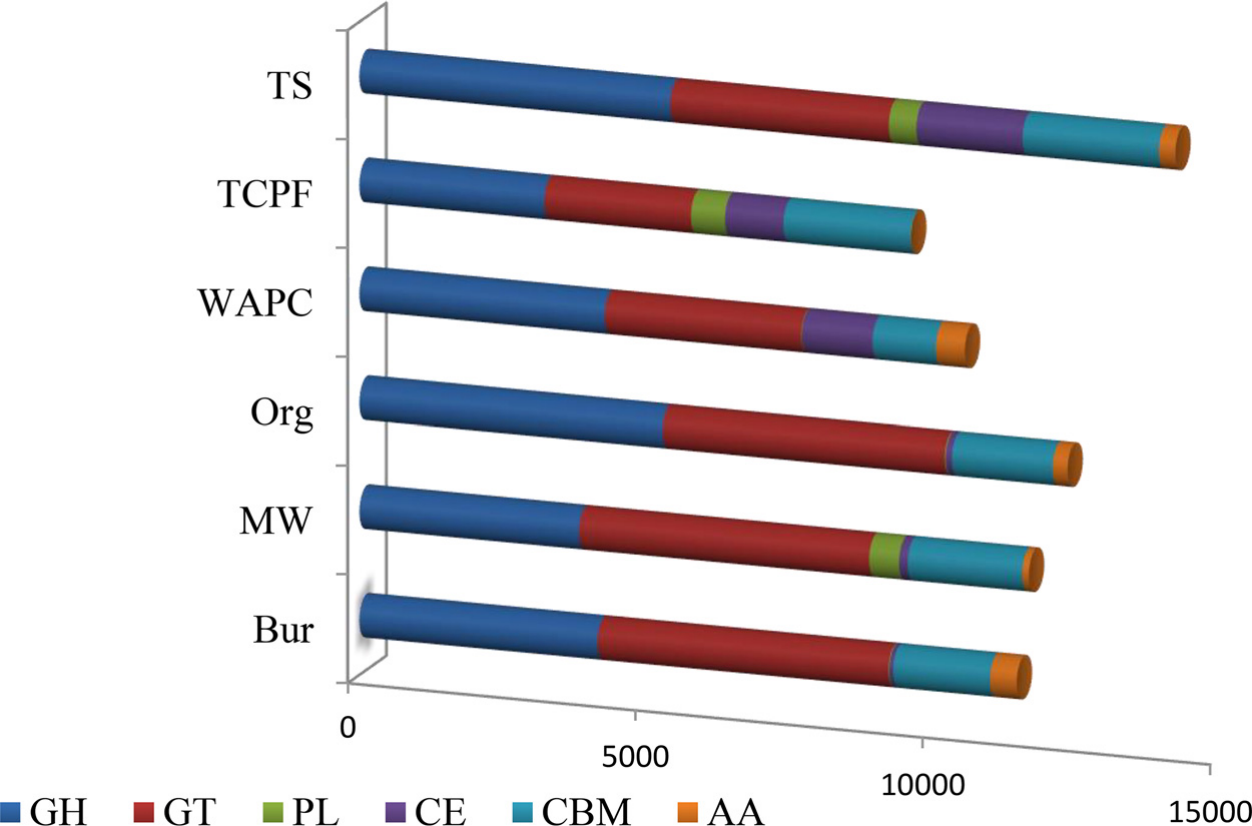
Carbohydrate-active enzymes in six samples. GH, glycoside hydrolase; GT, glycosyltransferase; PL, polysaccharide lyase; CBM, carbohydrate-binding module; AA, auxiliary activities; CE, carbohydrate esterases.

The number of genes belonging to the six classes of carbohydrate enzymes was determined by searching the unique genes against the Carbohydrate-Active Enzymes (CAZy) database. Figure 11 shows the distribution of carbohydrate enzymes in the six samples. A high number of glycoside hydrolase (GH) genes were identified in the TS, TCPF, and WAPC samples, and class auxiliary activity (AA) genes were at the lowest numbers. Moreover, glycosyltransferase (GT) genes were predicted to be predominant and class carbohydrate esterase (CE) genes were observed to be the lowest in the Org, MW, and Bur samples. The polysaccharide lyase (PL) gene was found to be present only in the TS, TCPF, and MW samples.

**FIG 12:**
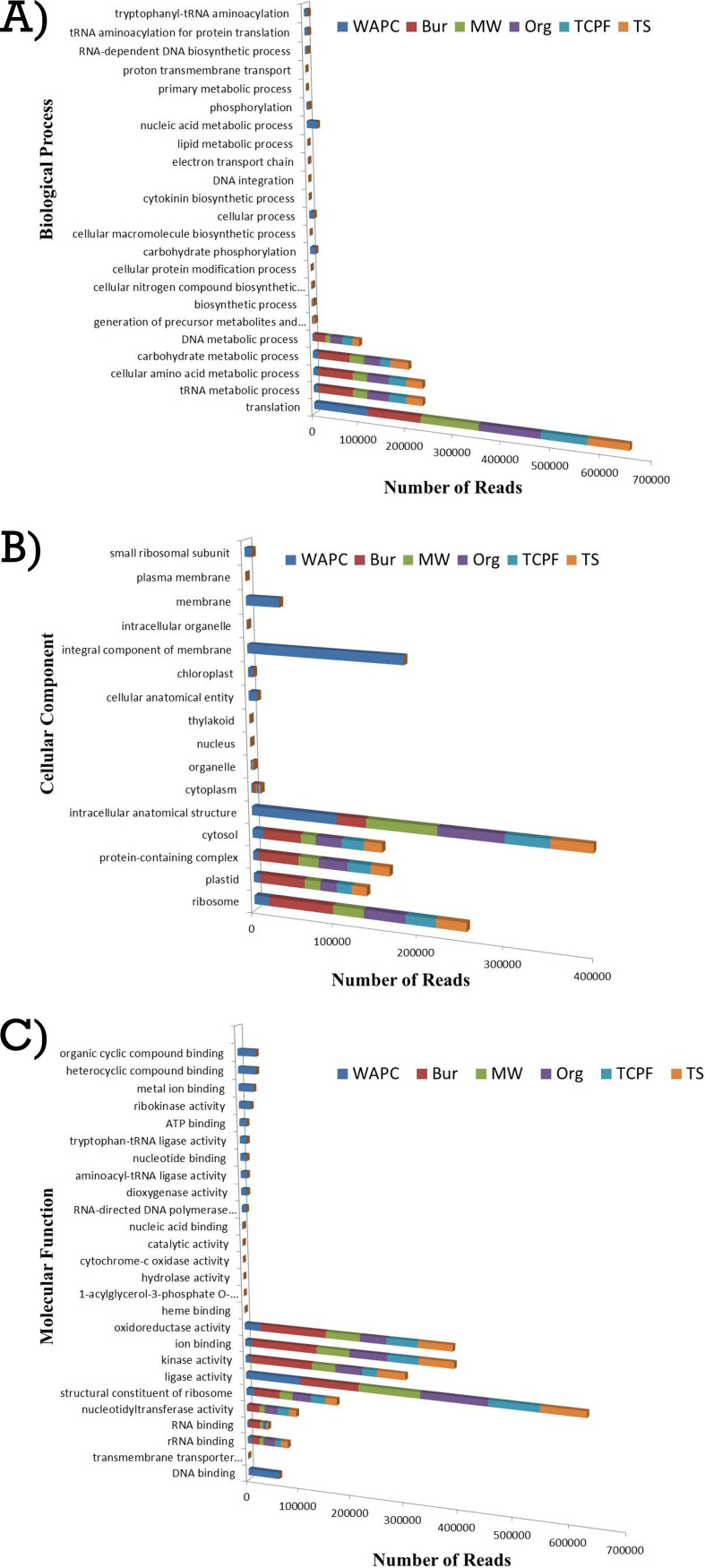
KEGG annotation pathway (A) Biological Process; (B) Cellular Component; (C) Molecular Function.

In gene ontology, the highest number of reads in the biological process (BP) was found to be associated with the translation process (the number of reads mapped in all samples varied from 83,324 to 126,772), followed by tRNA metabolic process (10,556 to 74,716) and cellular amino acid metabolic process (10,556 to 74,712) in all six samples (Fig. 12). In the cellular component (CC), reads were predominantly assigned to integral component of membranes (183,862) for the WAPC sample, whereas in the Bur sample, reads were abundant for the ribosome (79,130), followed by plastid (55,746), protein-containing complex (50,118), and cytosol (46,215). Moreover, almost similar reads were assigned to ligase activity in all six samples. In molecular function (MF), DNA binding, organic cyclic compound binding, heterocyclic compound binding, metal ion binding, ribokinase activity, and ATP binding were assigned only to reads of the WAPC sample. In the Bur sample, oxidoreductase activity, ion binding, and kinase activity were major MFs assigned to the reads. The highest number of reads was assigned to the pentose phosphate pathway in all six samples, followed by the aminoacyl-tRNA biosynthetic pathway.

**FIG 13:**
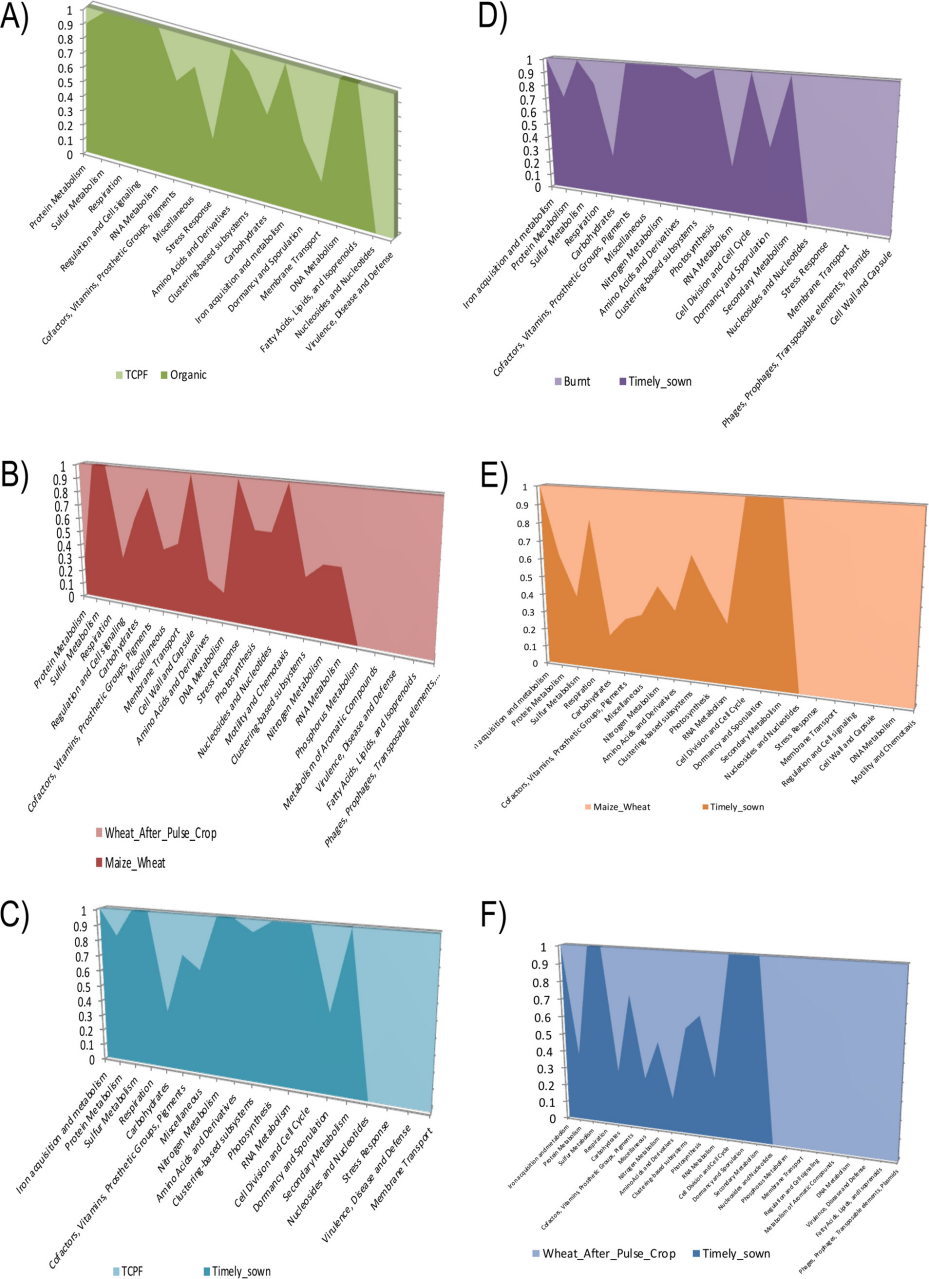
Subsystem analysis at functional level. (A) TCPF and organic; (B) maize-wheat and wheat after pulse crop; (C) timely sown and TCPF; (D) timely sown and Bur; (E) timely sown and maize-wheat; (F) timely sown and wheat after pulse crop.

To gain a better understanding of probable metabolic features, functional annotation was performed using the SEED (subsystem-based annotation) database with an 80% threshold. All subsystems in all comparison sets were clustered into different groups based on their functions. Carbohydrate metabolism was observed as the predominant subsystem. Clustering-based systems have been observed in organic, timely sown, and wheat after pulse crop samples, with protein metabolism in timely sown and organic samples. Subsystems such as amino acids and derivatives and cofactors, vitamins, prosthetic groups, and pigments were observed in samples such as wheat after pulse crop and maize-wheat (Fig. 13).

Table 2 contains the details of the identified microbial community along with the functions in relation to wheat (*Triticum* sp.). Table 3 describes the microorganisms specific to each sample under study with their relative abundance.

**TABLE 2:** Microbial community identified in the study with functions and references with respect to wheat (*Triticum* sp.)

Plant growth-promoting organism(s)	Source	Plant growth regulation^*a*^	Results of addition of bacteria to plants	Reference(s)
Pseudomonas sp.	Wheat	P solubilization, ACC deaminase, siderophores, IAA	Increased soil enzyme activities, total productivity, nutrient uptake, nutrient assimilation	40
*Bacillus* spp.	Rhizospheres of wheat and tomato	IAA, lipase, protease, siderophore, P solubilization, salt tolerance, silicate and zinc solubilization, biofertilization for micronutrients	Germination, root length, root wt, panicle wt	32
*Bacillus* RC01	Rhizosphere of wheat	P solubilization, N_2_ fixation	Root and shoot wt, total biomass	58
*Bacillus* sp. AW1	Rhizosphere of wheat	P-solubilization, N_2_ fixation, ACC deaminase siderophore, ammonia, HCN	Seedling length, germination, plant ht, panicle wt, root wt	59
Bacillus firmus	Wheat roots		Biomass, number of ears, nitrogen accumulation, N content	60
Pseudomonas spp.	Rhizosphere of wheat	P solubilization, siderophore	Protein content, yield, and grain quality	41
*Bacillus* sp., Pseudomonas sp.	Wheat rhizosphere	P solubilization	Plant biomass	33
Acinetobacter lwoffii		N_2_ fixation, siderophores, P solubilization	Root growth	31
*Flavobacterium*, *Streptomyces*		Agriculture productivity		35 – 37
*Paenibacillus* sp., *Rhizobium* sp.	Wheat seed		Increase growth yield, biochemical content, and high pH tolerance	38
*Paenibacillus* sp.	Wheat	Cytokinin, N_2_ fixation	Plant growth	39

a ACC, 1-aminocyclopropane-1-carboxylate; IAA, Indole-3-acetic acid.

**TABLE 3:** Microorganisms specific to each sample and relative abundance^*a*^

Organism	Relative abundance in indicated sample					
	Bur	MW	Org	WAPC	TCPF	TS
Acinetobacter lwoffii	0.103696304	2.70216	0.00222598	0	1.533461	0.065553
Bacillus badius	0.992524008	0	0	0	0.673441	0
**Bacillus cereus**	0	2.400135	0	0	0	0
Bacillus firmus	1.7010045	0	3.017085	1.202745	2.505615	0.460607
Bacillus flexus	4.9215	10.998865	73.2232	15.9393	16.92455	16.6501
Bacillus foraminis	0	0	5.757	0.790366	1.41202	1.62968
**Bacillus horikoshii**	1.071634	0	0	0	0	0
Bacillus selenatarsenatis	2.91223	0	2.34982	0	1.541414	0
Flavobacterium columnare	0	1.25129575	2.183141	0	0	0.000274
Flavobacterium succinicans	4.784382359	9.8042655	51.11589	0	0	2.778055
Paenibacillus chondroitinus	0	0	7.646447	0	0	1.001457
**Pseudomonas alcaligenes**	0	0	1.14601	0	0	0
Pseudomonas nitroreducens	0	1.25750817	22.4461688	4.066081	0	0.473615
**Pseudomonas veronii**	0.5899255	0	0	0	0	0
**Streptomyces radiopugnans**	0	0.46068819	0	0	0	0

a Bold indicates unique microorganism observed in a single sample.

## DISCUSSION

Overall, this study focused on the diversified population of microbial communities from soil obtained from wheat agricultural field based on various conditions, including organic (Org), timely sown (TS), wheat after pulse crop (WAPC), temperature-controlled phenotyping facility (TCPF), maize-wheat (MW), and burnt field (Bur). Microbial diversity analysis at all levels indicated the presence of diversified microbial community populations in these samples. Based on the percentage of microbial populations from taxonomic summaries, it was observed that *Proteobacteria* was the predominant phylum in all the samples, followed by *Acidobacteria*. The phylum *Proteobacteria* includes many of the nitrogen-fixing bacteria that have symbiotic relationships with a few plant groups also (10) playing a major part in agriculture (11). The phylum *Acidobacteria* is described as enigmatic but still is known for its abundance distribution and ecological importance, including abilities in nitrogen fixation, response to soil nutrients, etc., thus alluding to its impact on agriculture soil (12). The presence of other phyla, including *Actinobacteria*, *Gemmatimonadetes*, *Bacteroidetes*, *Chloroflexi*, etc., was also observed. At the genus level, the highest relative abundance of the genera *Bacillus* and *Flavobacterium* was predicted for the TCPF and Org samples, respectively. The genus *Nitrospira*, which is reported to be the most abundant and ubiquitous group of nitrite-oxidizing bacteria (NOB), was observed in MW, WAPC, TS, and MW samples (5).

Acidobacteria are reported to help in soil recovery after drastic conditions, leading to enhance crop growth and nutrient cycling (13), and are also pollutant tolerant (14, 15). Their further subdivision into six classes are responders and tolerance of soil nutrients lead to the conclusion that its order o_iii1-15 might play a similar role in agricultural soil for the further study of the impact of the acidobacterial community on agricultural management (16). The overall identified microbial community at the species level in our study also supports the existence of varied organisms.

To better understand the significance of the diversity, a statistical analysis was performed. The Welch *t* test applied within two replicates for each sample at species level revealed a significant difference, with only organic sample replicates present at a significance level of <0.05. In addition, Fisher’s exact *t* test at the genus level estimated differences between populations and proportions in all comparison sets. For instance, in Bur and timely sown sample comparison sets, the microbial populations and proportions are completely different. In accordance with the observations from previous studies, bacterial communities in the Bur sample were enriched in *Actinobacteria*, *Bacteroidetes* (17), and *Chloroflexi* (18).

The abundant microbial community is pivotal and is discussed extensively in this report. The study also sought to determine whether the presence of rare species in the population had any significant effect. Study of the differentiation of the community revealed large populations of rare species. But these occurrences may be by chance, due to contamination with oil, like petroleum/industry waste/sewage, etc., or by use of biofertilizers. But despite these assumptions, keen examination of the rare species led the following conclusions.

Beneficial members of the microbial community in agriculture include phosphorus-solubilizing microorganisms, microbes with biocontrol properties such as neutralizing a variety of phytopathogens and insect herbivores, and organisms promoting bioflocculation and enzymatic activity. For instance, Paenibacillus macerans, classified as a rare species in all samples, is involved in all the above-listed activities (19–22). Microorganisms involved in enhancing crop productivity, substitutes to fertilizers and pesticides, include *Bacillus* and Pseudomonas species, which are found to have dominant roles (23). Others include the genera *Acetobacter*, *Burkholderia*, *Azospirillum*, Klebsiella, and *Serratia*, known as plant growth-promoting bacteria (24). Adverse effects of these rare species include intestinal contamination from pathogenic Klebsiella species, causing pneumonia, meningitis, etc. (25). The plant pathogen *Erwinia* causes diseases in plants that are economically important (26).

The activities within these microbial communities were analyzed based on their involvement in the overall functional class as well as subsystems. For the unknown protein coding ability, annotation and ribosomal category analyses were performed. It was revealed that most of the OTUs identified in samples are involved in carbohydrate subsystems; in-depth analysis may provide a better understanding of the pathways involved in carbohydrate metabolism, fixation, and so on (27). Our research results could provide an indication of the behavior of microbial composition under different conditions related to wheat grown in agricultural soils, the proportion of rare species, and whether their presence has positive or negative effects on crop growth.

Based on the results, at the phylum level, the wheat microbial community belonged to different taxonomic positions, mostly *Actinobacteria*, *Acidobacteria*, *Proteobacteria*, *Gemmatimonadetes*, and *Bacteroidetes*. While most of these phylum abundances were reported in a previous study (28) on the wheat microbiome, few of the phylum abundances in our study, including GN04, *Chloroflexi*, *Chlorobi*, *Nitrospirae*, and WS3 are yet not reported. Each of these phyla is reported to have an impact on wheat growth; for example, *Chloroflexi* is reported to play an active role in sediment carbon cycling and fixation, respiration of sugars, and fermentation (29).

The microbes identified in our study have potential roles with respect to wheat such as productivity, health, components like carbon, nitrogen fixation, phosphorus solubilization, resistance to diseases, and so on. Acinetobacter lwoffii is relatively more abundant in MW (2.7%) than in all other samples. It is an endophytic bacterium that benefit plants by their colonization and acts as a barrier against pathogenic microbes (30). It plays role in plant growth regulation through nitrogen fixation, siderophores, root growth, and phosphorus solubilization (31). *Bacillus* species like B. badius (Bur, 0.9%, and TCPF, 0.6%), B. firmus (Org, 3%; TCPF, 2.5%; Bur, 1.7%; and WAPC, 1.2%), B. flexus (highly expressed in Org [73%] and TCPF, WAPC, and TS [all 16%]; MW, 10.9%; and Bur, 4.9%), B. foraminis (Org, 5.7%; TS, 1.6%; TCPF, 1.4%; and WAPC, 0.8%), and B. selenatarsenatis (Bur, 2.9%; Org, 2.3%; and TCPF, 1.5%) expressed in the samples are described, along with their relative abundances. Apart from the above *Bacillus* species list, there are few species that were very specific to a single sample only, which may be of significant interest. They are B. cereus and B. horikoshii, which were expressed only in the MW (2.4%) and Bur (1%) samples, respectively. All these *Bacillus* species have wider roles in plant regulation like phosphorus solubilization, zinc and silicate solubilization, salt tolerance, siderophore, nitrogen fixation, as producers of phytohormones like cytokinin, as biofertilizers for micronutrients, and as biocontrol agents against pathogens (28, 32, 33). Siderophore production activity has role in plant farm yard management. *B. horikoshii* is a rhizospheric microbe that helps in fixing carbon and nitrogen sources in the soil, which was seen at a significant level only in the Bur sample (34).

Flavobacterium columnare was observed in the Org (2%) and MW (1.2%) samples, while *F. succinicans* was highly expressed in the Org sample (51%) and was seen in other samples also, like the MW (9.8%), Bur (4.7%), and TS (2.7%) samples. Both of these *Flavobacterium* species were not expressed significantly in the WAPC and TCPF samples. *Flavobacterium* is a wheat rhizospheric microbe that plays a role in overall agricultural productivity (35–37). Streptomyces radiopugnans was expressed significantly in only the MW sample (0.4%) and function in a manner similar to that of *Flavobacterium* species in agricultural productivity (35–37). Paenibacillus chondroitinus was seen in the Org (7.6%) and TS (1%) samples. It helps in increasing growth yield, high pH tolerance, nitrogen fixation, and cytokinin production (38, 39).

Pseudomonas nitroreducens was detected in the Org (22%), WAPC (4%), MW (1.2%), and TS (0.5%) samples. However, P. alcaligenes and P. radiopugnans were recorded in the Org (1%) and Bur (0.5%) samples, respectively. Pseudomonas species plays a wider role in soil enzyme activities, nutrient regulation (including assimilation and uptake), total productivity (like crop protein content, yield, and grain quality), and plant biomass (33, 40, 41). It also aids in the production of phytohormones such as auxin, which regulate shoot elongation and related plant physiological processes (42). P. alcaligenes plays a role in the biological control of Fusarium wilt on lentils (43).

### Conclusion.

This study gives us a deeper insight to unveil the wheat microbiome in terms of structural diversity, composition, and plant growth functional genes under different agricultural practices, namely, Org, TS, WAPC, TCPF, MW, and Bur. The outcomes of this research work suggest the dominance of the phyla *Proteobacteria*, *Acidobacteria*, *Actinobacteria*, and *Gemmatimonadetes*. We found abundances of *Actinobacteria* and *Proteobacteria* in the TCPF sample, *Bacteroidetes* and *Gemmatimonadetes* in the Org sample, *Acidobacteria* in the MW and WAPC samples, *Proteobacteria* and GN04 in the Org sample, and *Bacteroidetes* and *Chloroflexi* in the TS and Bur samples. The analysis based on phylum level revealed abundances of *Proteobacteria*, followed by *Actinobacteria*, *Acidobacteria*, *Proteobacteria*, *Gemmatimonadetes*, and *Bacteroidetes*, in majority in all the comparison sets, while the relative abundances varied at the genus level. The highest relative abundances of the genera *Bacillus* and *Flavobacterium* were predicted in the TCPF and Org samples; the genus *Nitrospira*, the ubiquitous nitrite-oxidizing bacterial group, had higher relative abundances in MW versus WAPC, TS versus MW, and TS versus WAPC samples. We also observed a few distinct phyla like GN04, *Chlorobi*, *Nitrospirae*, and WS3. We identified the genes belonging to the six classes of carbohydrate enzymes using the CAZy database, which revealed high numbers of glycoside hydrolase genes in the TS, TCPF, and WAPC samples. The glycosyltransferase gene was predominant while the carbohydrate esterase gene was the lowest in the Org, MW, and Bur samples. The polysaccharide lyase gene was present only in the TS, TCPF, and MW samples. Finally, functional annotation unveiled carbohydrate metabolism as the predominant subsystem. In conclusion, we observed that the crop sequence has significant influence on soil microbial diversity and community composition, signifying its economic and environmental value as a sustainable farming approach for safeguarding the food security and ecosystem health. The results of this study could help wheat researchers better understand these dynamics. Also, most microbes are not culturable, and microculture technology can be a potential approach to exploit other cultured microorganisms to increase throughput and result in unique species for further commercial production.

## MATERIALS AND METHODS

### Sample collection and data generation.

To study the soil microbial community, soil samples were collected from different wheat-growing screening locations within ICAR-IIWBR research farm, Karnal, India (29.6857° N, 76.9905° E), representing various growing conditions. These samples were collected from the standing wheat crop during the time of anthesis, up to a depth of 15 to 20 cm from the soil surface, using an auger slanting toward the root zone from the interrow space. Two samples from either side of the wheat crop row were drawn. The four samples were mixed properly to draw a final sample for DNA extraction from each condition separately. These were further transported on ice to be stored at −20°C for immediate analysis in the lab.

The soil samples were collected in duplicates categorized into organic (Org; fixed plot maintained by using farm yard manure (FYM) alone as the source of nutrients), timely sown (TS; the crop was timely sown), wheat after pulse crop (WAPC; wheat grown after pigeon pea, which is a pulse crop), temperature-controlled phenotyping facility (TCPF; higher temperature, i.e., 5°C above ambient temperature, was maintained particularly during the postvegetative wheat crop stage), maize-wheat cropping system (MW; wheat grown after the harvest of maize in the month of November), and residue burnt field (Bur; residue of rice was burnt under controlled conditions prior to planting wheat). Total DNA extraction from soil samples was performed by using the MO-BIO PowerSoil DNA isolation kit, following manufacturer instructions, and the purity of isolated DNA was checked by measuring absorbance at 260 and 280 nm.

### Preprocessing of raw data.

Raw samples with pair-end reads were preprocessed using the Fastq-join method (44). All unpaired reads containing any uncertain bases were filtered out. This was followed by demultiplexing based on a barcode sequence; quality trimming was executed with a quality Phred score of ≥20, trimming the N-character reads, bases with bad quality having errors in barcode.

### Microbial community analysis.

**(i) Sequence analysis.** The Quantitative Insights Into Microbial Ecology (QIIME) bioinformatics pipeline was used for analyzing the microbial community composition of 16S rRNA gene Illumina sequence data (45).

**(ii) Upstream sequence analysis.** During upstream analysis, preprocessed sequences were mapped into operational taxonomic units (OTUs) using the UCLUST algorithm (46). The open reference selection method was implemented for aligning the representative sequences against the Greengenes reference database (47). Each OTU representative sequence was subjected to taxonomic classification using the RDP (Ribosomal Database Project) naive Bayesian classifier (48). The FastTree method (49) was used for phylogenetic tree construction.

**(iii) Downstream sequence analysis.** The in-built Perl script was used to calculate the relative abundance level of each taxon in the samples. Weighted and unweighted UniFrac distances (50) were calculated for identified OTUs in all samples. Alpha diversity indices were computed using Simpson-Shannon species diversity. For species-level analysis, Spearman’s rank correlation coefficients were used to generate a heat map (51). Compositional similarity between samples was analyzed with the Bray-Curtis score for taxonomic similarities through a nonmetric multidimensional scaling plot using PASTv3.11 software (45, 52). The predicted phylogenetic tree was visualized as a phylogenetic cladogram using MEGAN software (53).

### Functional analysis and annotation.

Gene ontology and pathway analysis with the Kyoto Encyclopedia of Genes and Genomes (KEGG) was performed using the DIAMOND tool (54). FragGeneScan 1.31 was used to analysis of protein-coding regions in sample reads (55). The Carbohydrate-Active Enzymes (CAZy) database was downloaded to perform a local BLAST search against the protein-coding regions identified with FragGenScan 1.31 with version NCBI-BLAST-2.9.0+ (56, 57).

### Data availability.

The data of the wheat microbiome metagenomics study under different agricultural field conditions have been submitted in the NCBI repository with BioProject number PRJNA794312. BioSample numbers are as follows: SAMN24624467 (organic [TaxID 410658]), SAMN24624468 (maize-wheat [TaxID 410658]), SAMN24624469 (timely sown [TaxID 410658]), SAMN24624470 (wheat after pulse crop [TaxID 410658]), SAMN24624471 (TCPF [TaxID 410658]), and SAMN24624472 (residue burnt field [TaxID 410658]). SRA numbers are as follows: SRR17441639, SRR17441640, SRR17441641, SRR17441642, SRR17441643, and SRR17441644.
